# Bone regeneration materials and their application over 20 years: A bibliometric study and systematic review

**DOI:** 10.3389/fbioe.2022.921092

**Published:** 2022-10-05

**Authors:** Xudong Zhang, Qianming Li, Zhengxi Wang, Wei Zhou, Linlin Zhang, Yingsheng Liu, Ze Xu, Zheng Li, Chen Zhu, Xianzuo Zhang

**Affiliations:** ^1^ Department of Orthopedics, The Affiliated Provincial Hospital of Anhui Medical University, Anhui Medical University, Hefei, China; ^2^ The First Affiliated Hospital of USTC, Division of Life Sciences and Medicine, University of Science and Technology of China, Hefei, China; ^3^ Department of Orthopedics, Anhui Provincial Hospital, Wannan Medical College, Hefei, China; ^4^ Department of Orthopedics, Union Hospital, Tongji Medical College, Huazhong University of Science and Technology, Wuhan, China

**Keywords:** bibliometric analysis, bone regeneration material, drug delivery, extracellular matrix, hydrogel

## Abstract

Bone regeneration materials (BRMs) bring us new sights into the clinical management bone defects. With advances in BRMs technologies, new strategies are emerging to promote bone regeneration. The aim of this study was to comprehensively assess the existing research and recent progress on BRMs, thus providing useful insights into contemporary research, as well as to explore potential future directions within the scope of bone regeneration therapy. A comprehensive literature review using formal data mining procedures was performed to explore the global trends of selected areas of research for the past 20 years. The study applied bibliometric methods and knowledge visualization techniques to identify and investigate publications based on the publication year (between 2002 and 2021), document type, language, country, institution, author, journal, keywords, and citation number. The most productive countries were China, United States, and Italy. The most prolific journal in the BRM field was *Acta Biomaterialia*, closely followed by *Biomaterials*. Moreover, recent investigations have been focused on extracellular matrices (ECMs) (370 publications), hydrogel materials (286 publications), and drug delivery systems (220 publications). Research hotspots related to BRMs and extracellular matrices from 2002 to 2011 were growth factor, bone morphogenetic protein (BMP)-2, and mesenchymal stem cell (MSC), whereas after 2012 were composite scaffolds. Between 2002 and 2011, studies related to BRMs and hydrogels were focused on BMP-2, *in vivo*, and *in vitro* investigations, whereas it turned to the exploration of MSCs, mechanical properties, and osteogenic differentiation after 2012. Research hotspots related to BRM and drug delivery were fibroblast growth factor, mesoporous materials, and controlled release during 2002–2011, and electrospinning, antibacterial activity, and *in vitro* bioactivity after 2012. Overall, composite scaffolds, 3D printing technology, and antibacterial activity were found to have an important intersection within BRM investigations, representing relevant research fields for the future. Taken together, this extensive analysis highlights the existing literature and findings that advance scientific insights into bone tissue engineering and its subsequent applications.

## 1 Introduction

The osseous tissue, which is a living structure that serves multiple important functions in the body, has a strong ability to repair and regenerate after injury. However, its natural healing process is slow and lengthy. Moreover, the new tissue fails to achieve the original strength and structure in a short time period, resulting in physical and social disability of the patients (Gong et al., 2015). Trauma, infection, and failed surgery may all lead to the destruction of the bone structure and consequent function loss. Therefore, bone grafting or osteogenic biomaterials are usually required to accelerate the bone healing process while maintaining the quantity and quality of the bone mass.

Bone transplantation is a commonly used method for osteogenesis in clinical practice. Autologous, allogeneic, and new artificial bone materials are some commonly used bone implant materials. Autologous bone has good osteogenic, osteo-inductive, and osteoconductive properties, so it is the most successful material for repairing bone defects. Fibula, ilium, and ribs are all good bone grafting sites. However, autologous bone has limitations, such as prolonged operation time, insufficient bone supply, and infection. Allogeneic bone transplantation is a commonly used method of allogeneic bone transplantation in clinical practice. This transplantation can be divided into deep-frozen bone, freeze-dried bone, fresh allogeneic bone, and demineralized bone matrix (Pina et al., 2015; Iaquinta et al., 2019; Lee et al., 2019; Kačarević et al., 2020; Daneshmandi et al., 2021). However, allogeneic bone also has defects, such as immune rejection and increased transmission of infectious diseases. Most of the new artificial bone materials are composite materials. Synthetic materials include metal, bioceramic, and polymer chemical materials, among others. These materials have been widely used in bone regeneration. In recent years, applying nano-artificial bone materials has opened a new era of bone regeneration. The representative materials mainly include nano-hydroxyapatite (nHAP), alumina nano-compounds, nHAP/poly-L-lactic acid (PLLA), and other composite materials.

Tissue engineering is a promising approach in bone regenerative medicine. Bone tissue engineering implants precursor cells into biocompatible scaffolds combined with growth factors to form bone (Lanza, 2016; Birlea et al., 2017; Trohatou and Roubelakis, 2017; Borrelli et al., 2020). The scaffold provides structural support for the bone defect and can stimulate the body’s regeneration potential, promoting cell proliferation, migration, and differentiation in bone regeneration. Scaffolds have been widely used in combination with growth factors, autologous bones, and cells for tissue regeneration. In addition to scaffolds, BMPs, platelet-derived growth factor, and vascular endothelial growth factor can all act on stem cells and osteoblasts to induce bone regeneration at the defect site. MSCs are widely used in bone tissue engineering and have the ability of self-renewal, abundant proliferation, and multi-lineage differentiation. MSCs are easy to isolate, culture, and expand and can maintain multi-directional differentiation potential for a long time. Thus, their use in bone tissue engineering is increasing (Pina et al., 2015; Yu et al., 2015; Hasan et al., 2018; Lee et al., 2019).

Scientific publications are central to discipline development and academic exchange, as well as for the creation of clinical practice guidelines. Evaluative bibliometrics is a field of quantitative science that uses different methods to assess research performance. For example, citation data is used to quantify the impact of an article over time, as shown by the number of times the article is cited. Thus, bibliometric analysis can be used to identify influential articles that shape medical practice and promote new research ideas. Previous bibliometric studies have pointed out the most highly cited papers in the field of bone regeneration in recent years (Huang et al., 2020), and qualitatively or quantitatively described commonly used polymer materials (Hussin et al., 2021) and artificial extracellular matrices (Simmons et al., 2020) in the field of bone tissue regeneration. Although these studies have identified several classic references in the field of bone tissue regeneration, they focused mainly in the field of materials research, whereas a comprehensive perspective on the related medical concepts is still lacking. To close this knowledge gap, a bibliometric analysis was conducted to identify all relevant scientific papers published on the Institute for Scientific Information (ISI) network, and assess the research trends on bone regeneration materials and their applications over the past 20 years.

## 2 Materials and methods

The ISI Web of Science Core Collection (WOS), which includes the Science Citation Index Expanded and other citation indexes, was used to retrieve scholarly articles and literature related to bone regeneration materials (BRMs) that were published between 2002 and 2021. For this review, the keyword ‘mHealth’ was used, and the analyses and conclusions followed the recommendations of Preferred Reporting Items for Systematic Reviews and Meta-Analyses.

### 2.1 Systemic search strategy

A structured search on the WOS database was conducted using the following research strategy. First, ‘biomaterials’ and ‘bone regeneration’ terms were searched in the title, keywords, and abstract fields. From the obtained 1,758 literature citations, keywords were extracted, and statistical analysis was performed. The retrieved documents were exported in Bibtex format and documented using Endnote 20 desktop version (Clarivate, Philadelphia, PA, United States) (Gotschall, 2021). All documents were selected by two independent reviewers (ZXD and ZXZ).

### 2.2 Eligibility Criteria

**FIGURE 1:**
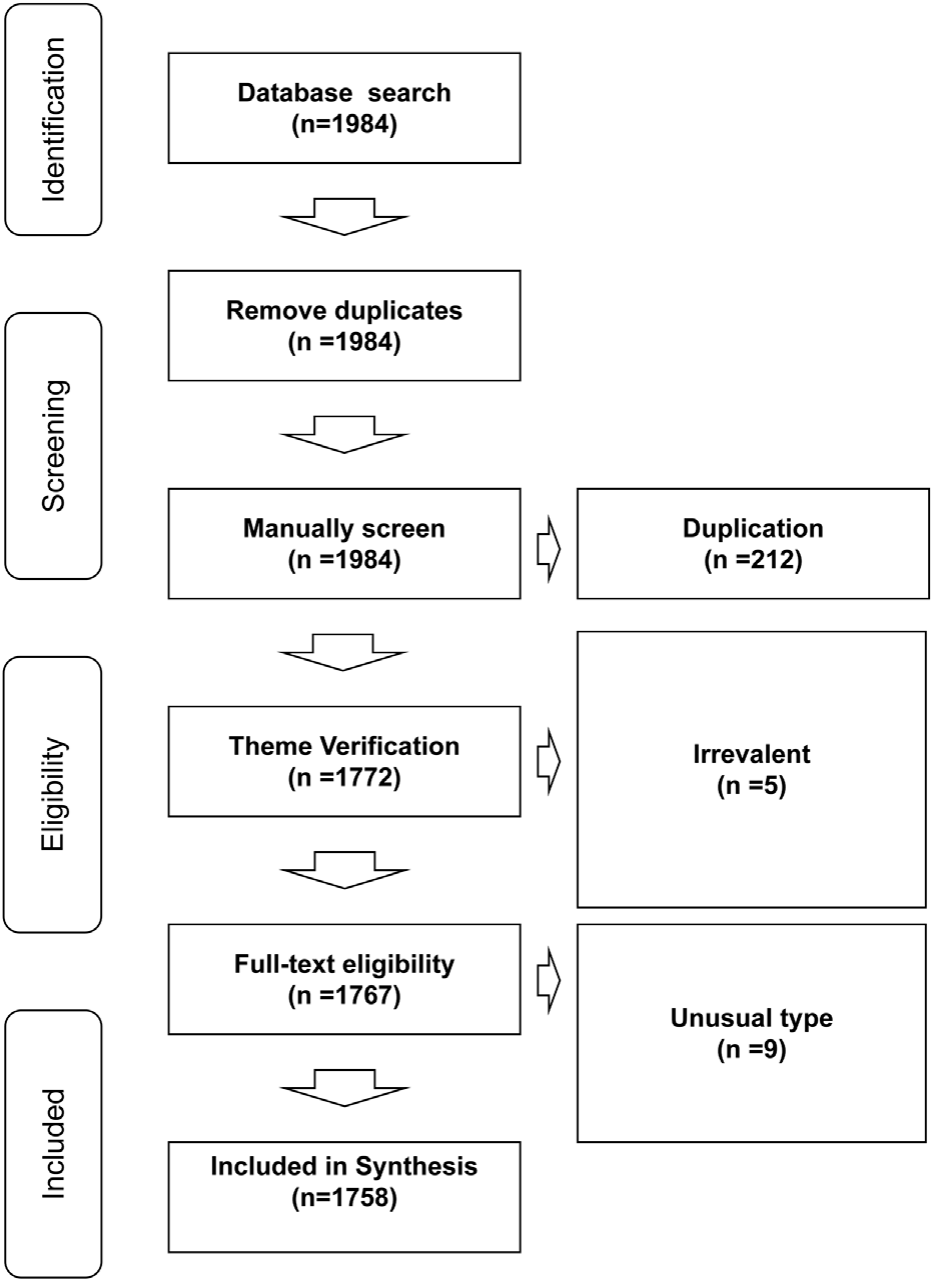
Criteria and flowchat for inclusion and exclusion of studies.

To be included in the study, the bibliographic documents had to agree to the following inclusion criteria: 1) be original/review articles, proceedings papers, book chapters, or other common types of publications retrieved by WOS; 2) had been published between 2002 and 2021 (including publications in various languages such as English, Chinese, French, Latin, Korean, among others); 3) report basic experiments, animal experiments, or clinical trial data on BRMs. Duplicated files, documents that did not covered BRMs, and publication files of uncommon categories (such as meeting abstract, early access, editorial materials) were excluded from the study. The inclusion/exclusion criteria applied in the present systematic review are shown in Figure 1.

### 2.3 Bibliometric Data Analysis

Citation metrics were assessed using Rstudio v4.1.3 software (2017-06-30; http://www.rstudio.com/) equipped with the *bibliometrix* package http://www.bibliometrix.org). Bibliometric networks and bibliographic coupling, including author, author keyword, citation, co-citation, collaboration, country, co-word analysis, keyword plus networks, and keyword co-occurrences, were visualized using *bibliometrix*. The H-index, G-index, and M-index calculated were limited to the corresponding subsets in the study field. The method of document processing and measurement is shown in Supplementary Figure S1. In addition to bibliometric data analysis, an in-depth reading of publications in many authoritative journals (including articles, reviews, proceedings, papers, etc.) was conducted. We summarized the research done in the field of bone regeneration based on three keywords, such as “ECM,” “hydrogel,” and “drug delivery” in the past 20 years, focusing on the most popular materials used, their osteogenic mechanisms, and their engineering applications in bone tissue. We also analyzed the application of scaffolds in osteogenesis based on the three searched topics or subsets. In addition, we also briefly discussed 3D printing and citrate research in bone tissue engineering.

## 3 Results

**FIGURE 2:**
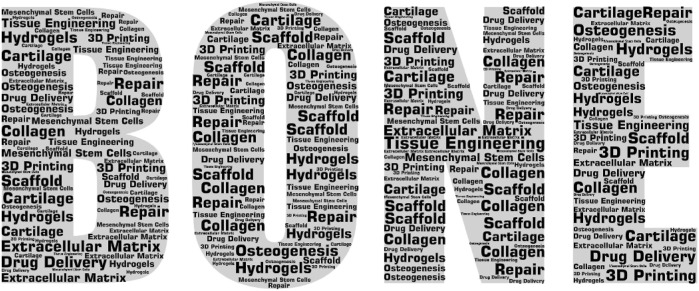
Wordcloud of the collection of literature keywords included in the study.

A total of 1,758 articles met the inclusion criteria that yielded various keywords (Figure 2) in the fields of ECMs (*n* = 298) and hydrogel (*n* = 269). It was also found that the number of articles published in the journals increased between 2002 and 2021, indicating a growing interest in and expansion of the bone regeneration research field. After manually filtering out the keywords that were irrelevant or meaningless to the subject of analysis, ‘ECM,’ ‘hydrogels,’ and ‘drug delivery’ were found to be high-frequency words. Therefore, the selected documents were further assessed according to these three subset keywords. In addition, due to changes in the research subject during the past 20 years, which was highlighted by visual analysis, the selected documents were divided in two groups (2002–2011 and 2012–2021) according to their publication date using the PUBYEAR keyword as filter.

### 3.1 Publication journals and article types

**FIGURE 3:**
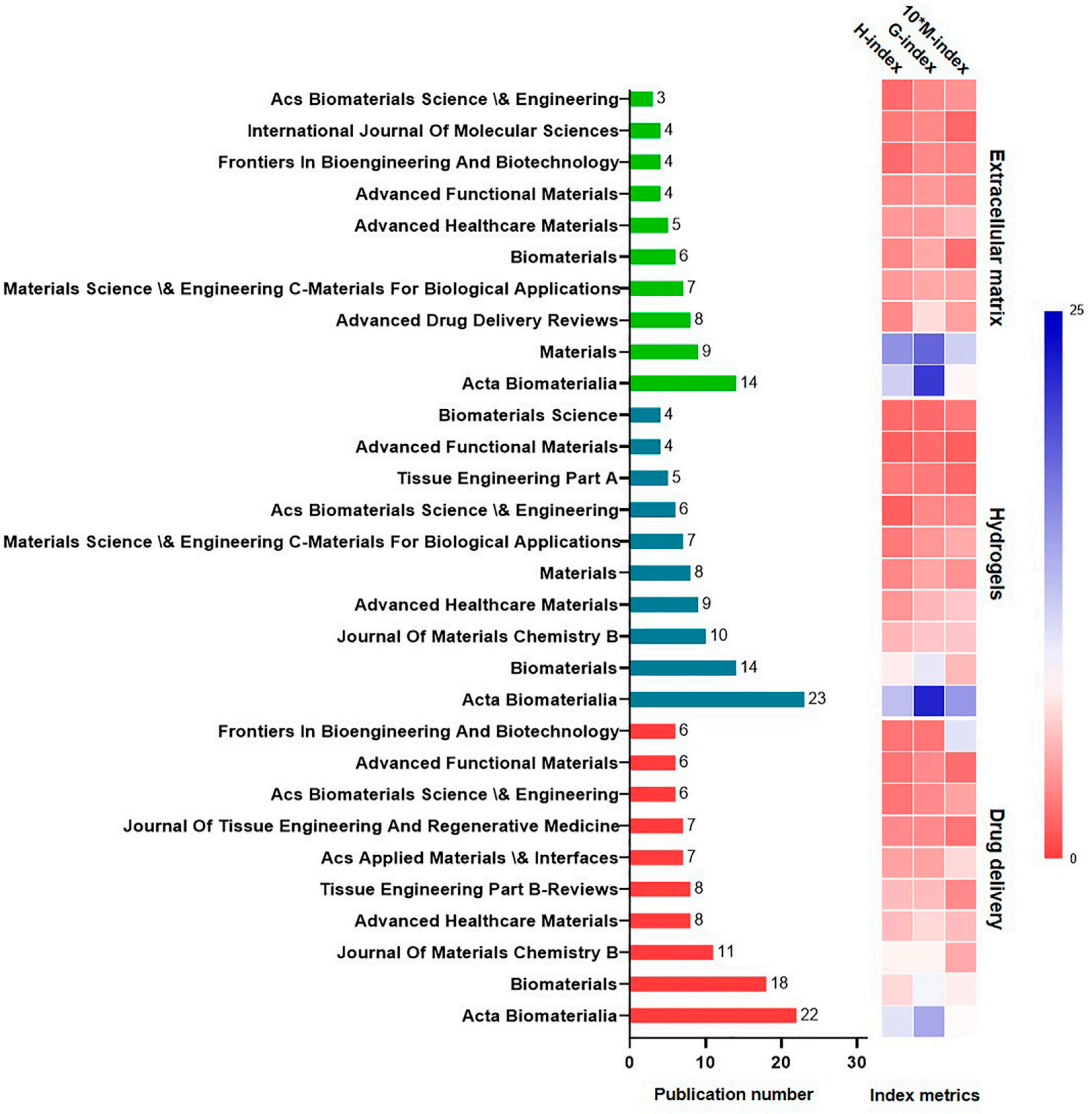
Publication number and index metrics for top 10 prolific journals.

The 10 professional journals with the most published papers in each subset are listed in Figure 3. The most prolific journal was *Acta Biomaterialia*, closely followed by *Biomaterials*. Overall, 96 articles were published in *Acta Biomaterialia*, 69 articles were published in *Biomaterials*, and 45 articles were published in the *International Journal of Molecular Science*.

Concerning publication type (Table 1), articles accounted for more than 50% of all publications. Analysis of these documents according the three most common keywords showed that 185 articles were related to the ECM, whereas 160 and 91 covered hydrogels and drug delivery systems.

**TABLE 1:** Publication types of selected research.

	Extracellular matrix (n = 370)	Hydrogels (n = 286)	Drug delivery (n = 220)
Article	185 (50%)	160 (55.94%)	91 (41.36%)
Review	164 (44.32%)	109 (38.11%)	115 (52.27%)
Proceedings paper	4 (1.08%)	4 (0.00%)	1 (0.45%)
Book chapter	8 (2.16%)	6 (0.61%)	5 (2.27%)
Other	9 (2.43%)	7 (0.61%)	8 (3.63%)

### 3.2 Most active authors and visualized analysis (authors, countries,keywords)

As in shown in Table 2, Rui L. Reis from I3Bs Research Institute on Biomaterials, Biodegradables and Biomimetics of UMinho (Braga, Portugal) published the most articles between 2002 and 2021, including more than 20 high-quality papers in the field of BRMs, and achieved the highest H-indexes in the hydrogels and drug delivery fields. The scholar who had most papers published in the ‘ECM’ subset was Cato T. Laurencin from the University of Connecticut (Storrs, CT, United States), who was found to work closely Kevin W. H. Lo, Hao-Min Lan, Karen M. Ashe, and Tao Jiang in the field of three-dimensional (3D) bioprinting technology.

**TABLE 2:** Most active authors, their publications and H-index.

	Extracellular matrix			Hydrogels			Drug-delivery	
	Pulications	H-Index		Pulications	H-Index		Pulications	H-Index
Laurencin Ct	8	7	Reis RL	7	6	Reis RL	7	5
Li X	6	5	Alsberg E	5	5	Vallet-Regi M	5	5
Reis Rl	6	5	Zhang X	5	4	Arcos D	4	4
Zhang Y	6	5	Jeon O	4	4	Chen FM	4	4
Kaplan Dl	5	5	Liu X	4	4	Kaplan DL	4	4
Wang Z	5	5	Wang H	4	4	Orive G	4	4
Zhang X	5	3	Wang X	4	4	Tabata Y	4	4
Chen Fm	4	4	Zhang Y	4	4	Wang Z	4	4
Chen X	4	2	Ameer GA	3	3	Webster TJ	4	4
Ghezzi Ce	4	4	Censi R	3	3	Baino F	3	3

**FIGURE 4:**
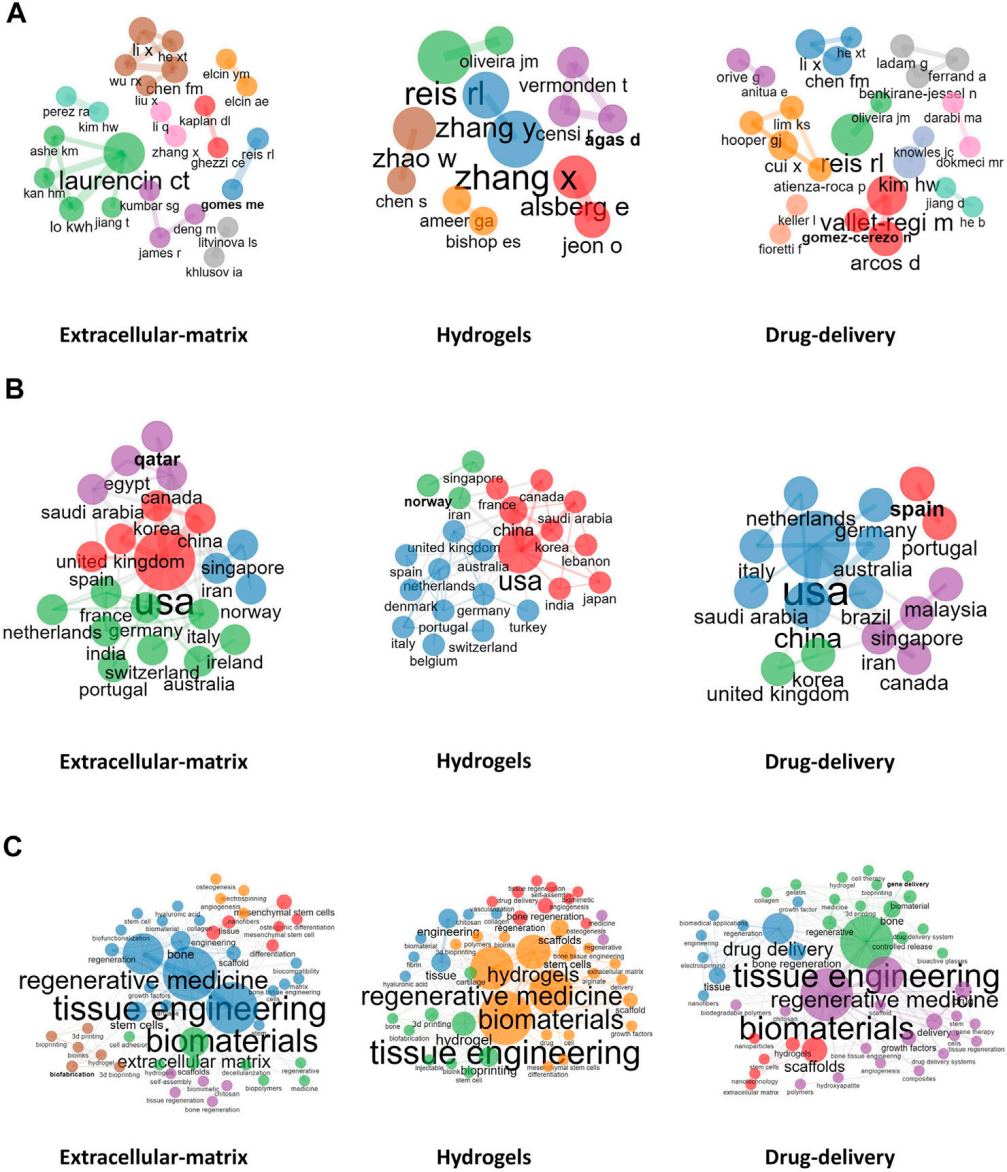
Visualized analysis. **(A)**: Collaboration between authors; **(B)**: Collaboration between countries; **(C)**: Collaboration between Author keywords.

Xiaoling Zhang from the College of Polymer Science and Engineering, Sichuan University (Chengdu, China) and Ying Zhang from the School of Chemical Engineering and Technology, Hebei University of Technology (Tianjin, China) were found to be leaders in the research of hydogels, being close collaborators in areas of nanocomposite adhesive hydrogels. Co-author visualization was used to illustrate the collaboration pattern of the authors (Figure 4A).

Concerning publishing countries, United States, China, Italy, South Korea, and Switzerland were found to have the highest total number of published articles. During 2002–2011, China published fewer papers, with 32, 34, and 38 papers published in the fields of ECM, hydrogels, and drug delivery, respectively. However, from 2012 to 2021, which marked a breakthrough in the research of BRMs in China, the number of reports published by Chinese teams in the above fields rose to 857, 699, and 665, respectively, surpassing those of teams from the United States and becoming the country with the most research publications in these fields. Noteworthily, although Switzerland-based teams did not publish the most articles, the average number of citations per report was the highest in 2002–2011 and 2012–2021. A network representing the collaboration between countries in the field of ECM, hydrogels, and drug delivery is shown in Figure 4B. China–United States, United Kingdom–United States, and United States–Korea collaborations ranked first concerning investigations on the ECM. The country distribution of published papers is shown in Table 3.

**TABLE 3:** Statistics of publication country related to bone regenerative materials between 2002-2021.

	Extracellular-matrix				Hydrogels			Drug-delivery	
	Publications	Total citations	Average article citations	Publications	Total citations	Average article citations	Publications	Total citations	Average article citations
**2002–2011**									
United States	139	6,195	177	123	4,545	162.3	69	2,960	174.1
CHINA	32	772	128.7	34	628	69.8	38	1,011	84.2
ITALY	56	551	42.4	14	46	15.3	8	221	73.7
SOUTH KOREA	41	430	61.4	20	178	44.5	24	414	82.8
SWITZERLAND	32	1,620	202.5	21	1,534	255.7	11	446	148.7
PORTUGAL	9	112	112	14	572	190.7	21	330	55
GERMANY	18	176	44	1	0	0	4	247	247
United Kingdom	5	143	71.5	12	1,072	268	4	0	0
FRANCE	8	184	61.3	15	221	73.7	6	72	72
CANADA	10	114	38	1	0	0	20	226	45.2
**2012–2021**									
United States	615	5,086	44.61	671	5,236	45.93	435	4,029	53.72
CHINA	857	4,305	26.57	699	4,450	31.79	665	5,148	37.85
ITALY	160	1848	54.35	104	530	25.24	72	453	26.65
SOUTH KOREA	197	1,411	36.18	179	893	24.14	164	1,208	35.53
SWITZERLAND	36	435	62.14	27	393	98.25	4	116	78.96
PORTUGAL	97	606	28.86	149	882	35.28	72	578	41.29
GERMANY	154	848	33.92	144	523	18.68	101	972	44.18
United Kingdom	65	685	71.5	121	819	30.33	39	257	28.56
FRANCE	60	579	46.5	48	487	33.91	64	214	21.40
CANADA	55	149	12.42	40	416	16.23	43	221	15.47

As mentioned above, the keywords of the published documents were extracted and counted. ECM, hydrogels, and drug delivery were found to be high-frequency words; thus, were used as subset variable names. In addition, other keywords were also commonly indicated in papers published in BRM-related fields, such as MSCs, osteogenesis, among others. Co-occurrence relationship between the most commonly used keywords were also assessed (Figure 4C), which showed the existence of five clusters characterized by the most commonly used keywords in the ‘ECM’ subset. The representative papers of each cluster were Badylak et al. (2009), Lutolf and Hubbell (2005), Discher (2009), Engler et al. (2006), and Murphy and Atala (2014). Moreover, the content described in these reports covered the relationship between several clusters centered on keywords such as “tissue engineering,” “MSCs,” “scaffold,” “osteogenesis,” and “3D printing.” For example, Badylak et al. (2009) showed that bioscaffolds composed of ECM can promote the remodeling of various tissues.

### 3.3 Time distribution measures for publications and thematic evolution

**FIGURE 5:**
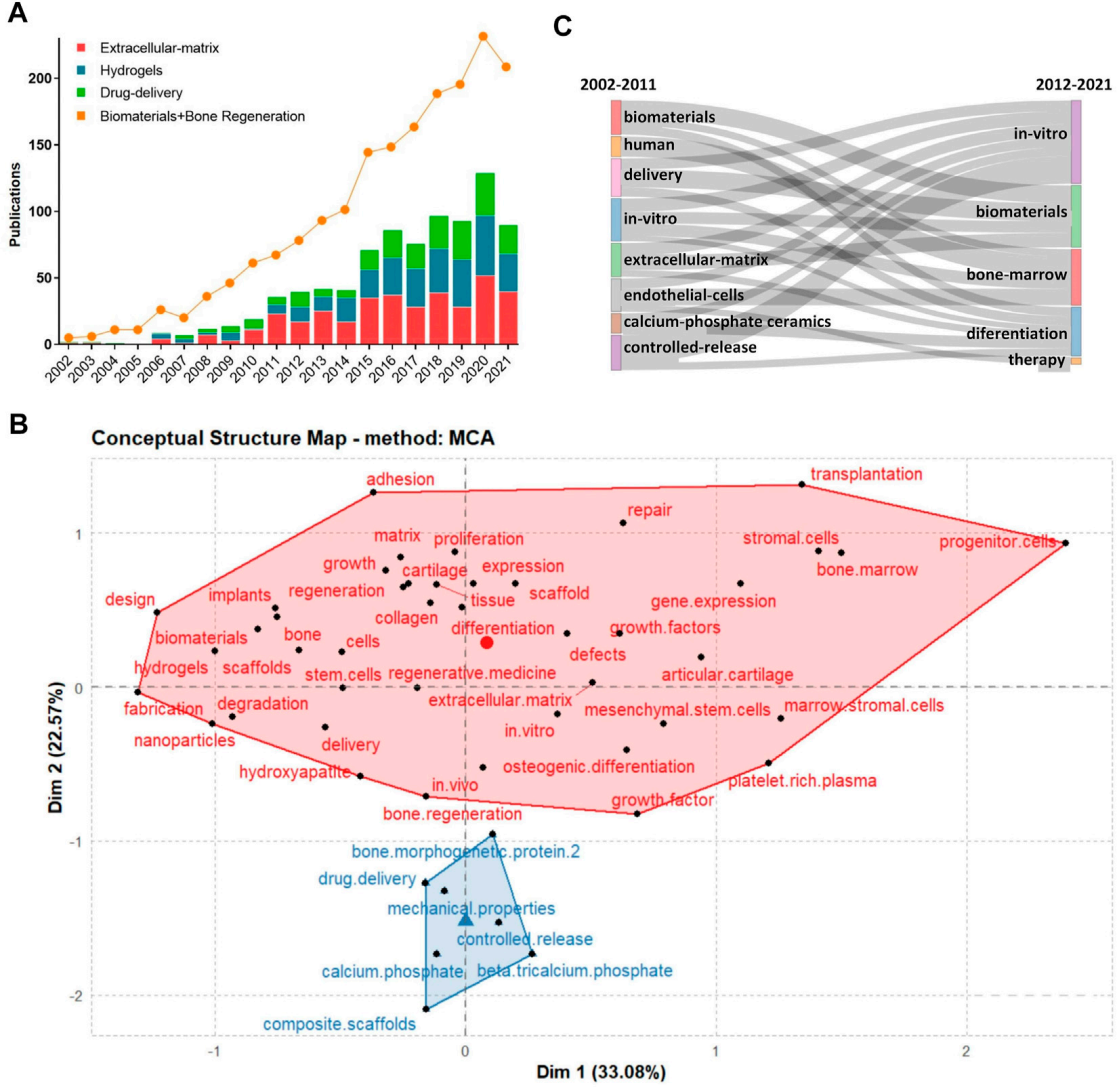
**(A)**: Study and publication trends between 2002–2021; **(B)**:Concept structure map of author keywords; **(C)**:Thematic evolution of studies during 2002–2021.

Between 2002 and 2021, the number of publications related to BRMs increased exponentially (Figure 5A). Specifically, under the ‘ECM’ subset, a total of 370 articles were published from 2002 to 2021, but only single-digit articles were published each year before 2009. The turning point occurred in 2010, with the publication of the article “Chondrogenic mRNA expression in prechondrogenic cells after blue laser irradiation” (18 citations to date) by Kushibiki et al. (2010), which opened a new research era on the ECM. In 2020, the number of studies using “ECM” as keyword reached its peak, with a total of 52 articles published. Hydrogels have been a hot topic in the field of biological tissue engineering research, with the number of articles related to hydrogels increasing yearly between 2011 and 2020. The first literature on bone regeneration materials with ‘hydrogel’ as keyword was published in 2006 (108 citations to date). As a long-term research hotspot in material chemistry, drug delivery can empower relatively inert bone regeneration materials, as well as improve the speed and promote the direction of tissue regeneration. Research on drug delivery targeting bone regeneration was firstly published before 2002, and has generally maintained an upward trend over the past 20 years. The publication trends related to the above subject terms are shown in Figure 5A.

We also performed a co-word analysis of the co-occurrence of keywords, aiming at representing the conceptual structure of a framework. The conceptual structure map was created using the Multiple Correspondence Analysis method, which allows to determine the association between two or more qualitative variables. The map revealed two clusters, of which one (in red) had the most meaningful keywords, which means that the researchers were highly focused on the BRMs field (Figure 5B).

From 2002 to 2011, investigations on BRMs covered varied subjects and topics, among which “delivery”, “*in vitro*,” “controlled release,” and “ECM” were of particular interest. During the same period, some scholars focused on topics such as “endothelial cells” and “calcium-phosphate ceramics.” From 2012 to 2021, authors who originally studied “controlled release,” “ECM,” “endothelial cells,” and “calcium phosphate ceramics” had turned their attention to “*in vitro*” and “therapy” focused investigations (Figure 5C).

## 4 Discussion

Bibliometric analysis is a meaningful evaluation method that can be used to reflect research status and trends. Based on publications publicly available in the WOS database, this study provides the first comprehensive analysis of BRMs. The analysis also serves as a way to rank journals, institutions, and universities worldwide. The field of research related to biomaterials for bone regeneration is constantly evolving, which is reflected by the increased number of publications and the expansion of the research fields covered throughout the years. Trends over the past decade reflect the growing number of terms associated with clinical topics, such as ECM, hydrogels, drug delivery, bone marrow-derived MSCs, BMP-2, *in vitro*, and others.

### 4.1 Research on extracellular matrices and applications of scaffolds in osteogenesis

Subset 1 mainly focused on ECM research in the field of BRMs. Relatively few studies are available regarding this topic from 2002 to 2011. During the last 10 years, researchers have been interested in 1) bone ECM composition and integrins and 2) the relationship between ECM mimetic peptides and osteogenesis. After 2012, ECM research focused more on scaffolds, such as acellular extracellular matrix scaffolds and 3D electrospun nanofiber scaffolds.

#### 4.1.1 Bone extracellular matrices composition and integrins

Different ECM molecules can differentially regulate cell differentiation by interacting with specific cell receptors (Aplin et al., 2002). The ECM in the bone consists primarily of an organic phase and a mineral phase, which includes non-collagenous components, type I collagen, and other minor collagens. Non-collagenous proteins include proteoglycans, such as hyaluronic acid, decorin, and versican, as well as osteonectin, osteopontin, osteocalcin, fibronectin, and vitronectin (Shekaran and Garcia, 2011). The mineral phase of consists of the calcium phosphate compound hydroxyapatite. Integrins are a ensemble of receptors that promote cell binding to extracellular matrix proteins (Hynes, 2002). Studies have found that the α2β1 integrin is highly expressed in osteoblast-like cells and is also the main adhesion receptor used by these cells to adhere to collagen. The α2β1 integrin is related to the osteogenic pathway (Gronthos et al., 1997). Furthermore, the α2β1-mediated adhesion of mouse MC3T3-E1 pre-osteoblasts to type I collagen was found to activate Runx2/Cbfa1, a transcription factor that promots osteoblast differentiation (Takeuchi et al., 1997; Xiao et al., 1998; Xiao, 2000; Tamura et al., 2001). Several other integrin isoforms, such as α5β1 and αvβ3, have also been shown to regulate osteogenesis (Moursi et al., 1997; Xiao et al., 1998; Keselowsky, 2005; Martino et al., 2009; Hu et al., 2010).

#### 4.1.2 Relationship between extracellular matrices mimetic peptide and osteogenesis

Full-length native ECM polymers include collagen, fibrin, hyaluronic acid, acellular matrix, and bone sialoproteins (Caiazza, 2000; Perka, 2000; Karp et al., 2004; Rammelt et al., 2006; Ben-Ari, 2009). In recent years, gels, cross-linked membranes, and demineralized bone particles have been widely used for osteogenesis (Suckow et al., 1999; Kim et al., 2007; Kurkalli et al., 2010). ECM-derived peptides can circumvent the limitations of full-length native ECM polymers that are difficult to modify and control. FHRRIKA as an ECM-derived peptidehas the ability to promote osteoblast differentiation. It is a heparin-binding sequence found on many ECM proteins (Rapuano et al., 2004; Dettin, 2006; Dettin et al., 2009). Martino et al. also found that the engagement of FNIII9-10 and α5β1 may enhance osteogenesis.

#### 4.1.3 Decellularized extracellular matrix scaffolds

**FIGURE 6:**
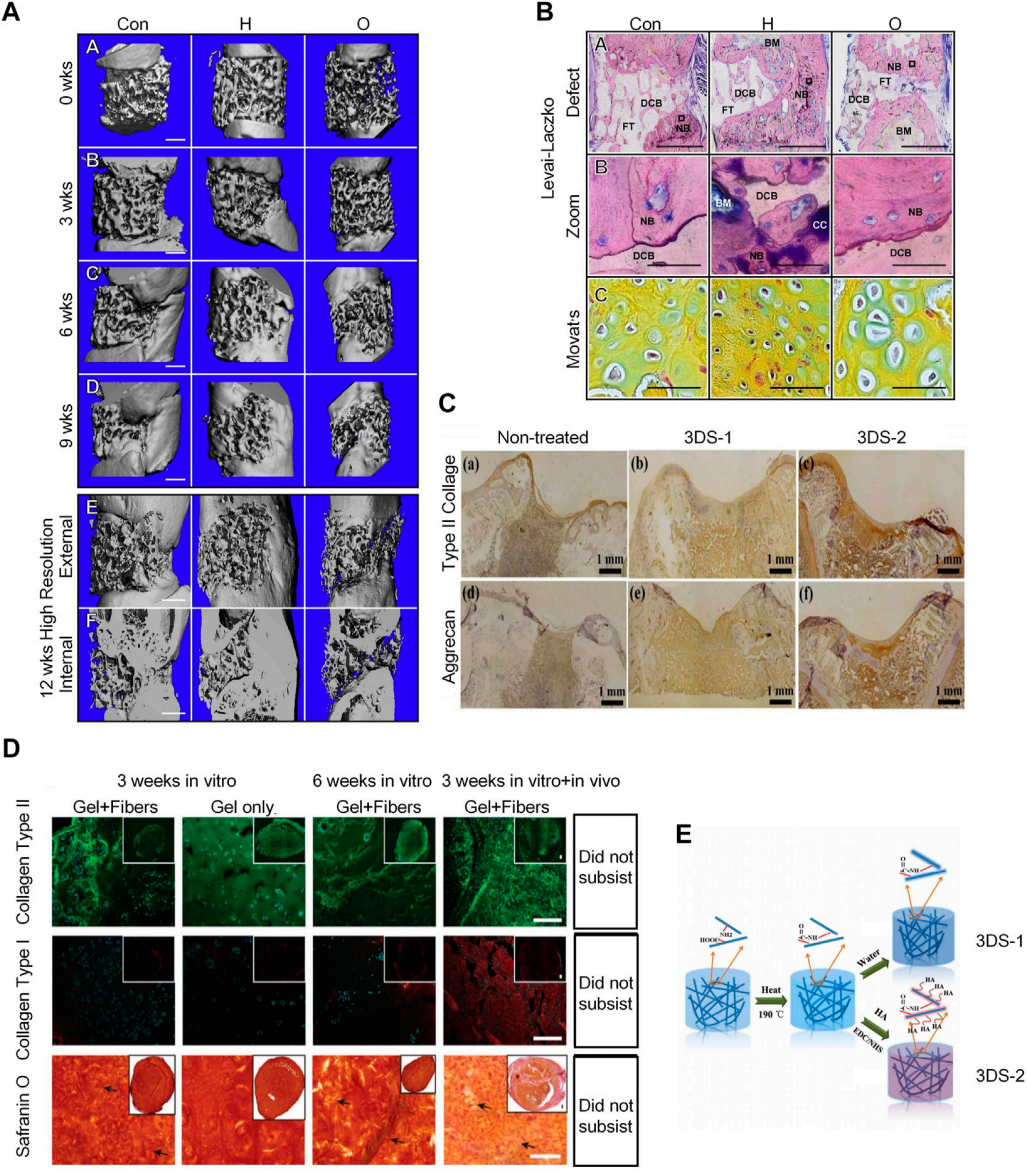
Application of dECMs and 3D Electrospun Nanofibrous Scaffolds in osteogenesis. **(A)** 3D μCT reconstruction of rat femurs with decellularized scaffolds, hypertrophic chondrocyte and osteoblast grafts at 0, 3, 6, and 9 weeks post-implantation to bridge severely sized femoral defects. The inner and outer areas are shown at 12 weeks. Defect regeneration and bone formation were seen 12 weeks after implantation. (Bernhard et al., 2017)(Reprinted with permission) **(B)**. Histology of hard bone using Levai-Laczko staining. The magnified view allows detection of calcified cartilage. At the site of new bone formation, there is cartilage primordium characteristic of endochondral ossification (green staining in Movat pentachrome sections) (Bernhard et al., 2017) (Reprinted with permission) **(C)**. Immunohistochemical staining results of type II collagen and aggrecan of untreated 3DS-1 and 3DS-2 scaffolds 12 weeks after implantation *in vivo*. (Chen et al., 2016) (Reprinted with permission)**(D)**. The hydrogel/nanofiber composite structures exhibited better chondrogenic ECM deposition and higher stability than pure hydrogel scaffolds in in vitro cell culture and *in vivo* implantation. (Chen et al., 2016) (Reprinted with permission) **(E)**. Schematic illustration of fabrication and cross-linking of electrospun nanofibrous porous 3D scaffolds (3DS-1) and hyaluronic acid scaffolds (3DS-2) cross-linking. (Chen et al., 2016) (Reprinted with permission).

The dECM scaffold is a three-dimensional framework mainly composed of ECM, mainly containing fibronectin, collagen, laminin, elastin, matrix cell proteins, etc (Chen and Liu, 2016; Liu et al., 2017a). Bernhard et al. also demonstrated that tissue-engineered bone grafts using dECM scaffolds can regenerate bone through endochondral ossification (Hesse et al., 2010; Scotti et al., 2010) (Figure 6A). They prepared decellularized bone scaffolds and used them for the treatment of rat femoral defects. The scaffolds were infused with adipose stem cells and cultured in a chondrogenic medium for 2 weeks, followed by a hypertrophy medium for 3 weeks to form hypertrophic cartilage. The cell-seeded structure showed excellent bone regeneration characteristics after implantation (Bernhard et al., 2017) (Figure 6B).

#### 4.1.4 Three-dimensional electrospun nanofibrous scaffolds

Electrospun nanofibers have a similar morphology to the ECM and possess properties for regulating cellular behavior and function; thus, they are frequently used in tissue engineering. 3D electrospun nanofibrous scaffolds can provide osteoblast with biomimetic fibrous structures and 3D microenvironments; therefore, they are good materials for tissue regeneration engineering. (Figure 6C) 3D ENF-S is classified into the following, according to different fabrication methods: 1) electrospun nanofibers created via direct electrospinning through post-processing techniques and tuning fiber collection techniques (Subramanian et al., 2011); 2) electrospun nanofibers/hydrogel composite 3D scaffolds fabricated by embedding the assembled electrospun nanofibers in a hydrogel precursor solution (Xu et al., 2010), and 3) electrospun nanofibers/porous matrix composite 3D scaffolds fabricated via 3D printing. 3D printing is a common method to fabricate porous scaffolds with complex structures, wherein the structural size and scale of electrospun nanofiber pores gradually increase from nanoscale to macroscale, similar to the topographical features of ECM (Lee et al., 2017). 3D ENF-S with a porous structure can also be easily formed by freeze-drying, which offers good stability and mechanical properties. In recent years, electrospun nanofibers have been widely used in cartilage tissue engineering. Chen et al. prepared gelatin/PLA nanofiber-based 3D porous scaffolds via freeze-drying and heating, then used 1-ethyl-3-(3-dimethylaminopropyl) carbodiimide (EDC)/N-hydroxyl succinimide (NHS) to cross-link the scaffold with hyaluronic acid (Figure 6D), which can ultimately further promote cartilage regeneration (Chen et al., 2016). A team also developed a double-layer collagen/PLLA nanofiber composite scaffold for bone tissue regeneration (Figure 6E). MSCs cultured as described above had a stronger osteogenic differentiation ability (Zhang et al., 2013).

### 4.2 Application of hydrogels in osteogenesis

Documents related to subset 2 were mainly associated with the use of hydrogels in the field of BRM. There were a few studies on hydrogels in the field of bone regeneration from 2002 to 2011. Most scholars have devoted themselves to studying the synthesis of hydrogels which can promote the differentiation of MSCs. Since 2012, research on hydrogels in the field of bone regeneration has been in full bloom. In recent years, researchers have been interested in hydrogels with photothermal effects, conductive hydrogels, and biomimetic self-assembled peptide hydrogels.

#### 4.2.1 Hydrogel-induced differentiation of mesenchymal stem cell

Native hydrogels can be used for regeneration and bone tissue repair; these can thus provide clues for inducing the differentiation of MSCs. However, some hydrogels have limitations in fine-tuning mechanical properties, leading to the consistent formation of gels with similar properties. Mauck et al.(2006) compared the growing of bone tissue that differentiated from MSCs in gels with that of native tissue. The results revealed that differentiated MSCs generate much less cartilage matrix than native cartilage tissue. Synthetic hydrogels provide materials with more easily controlled and reproducible properties. Polyethylene glycol (PEG) or poly (vinyl alcohol) (PVA), being the most widely used synthetic hydrogels for encapsulating MSCs, can be easily fine-tuned by changing the crosslink density as well as the expansion and compressive moduli of the system (Burdick, 2002). These have been shown to successfully generate an osteoid matrix with encapsulated osteoblasts. However, MSC encapsulation in these types of hydrogels may render cells unable to survive (Bryant et al., 2003; Nuttelman et al., 2005).

#### 4.2.2 Development of hydrogels for 3D culture of mesenchymal stem cell

From 2002 to 2011, good progress was made in developing hydrogel environments for the 3D culture of MSCs, which greatly helped in inducing MSC differentiation and matrix deposition. In addition, MSCs responded better to environments with a combination of TGFβ-1 and IGF-1 or BMP-6 than to those with either factor alone in directing cartilage matrix deposition (Indrawattana et al., 2004). BMP-2 and TGFβ-1 have synergistic effects on the chondrogenic differentiation of MSCs (Toh et al., 2005). It has also been shown that early delivery of FGF-2 prolongs the pre-osteoblast differentiation of MSCs in culture, while a much later delivery of BMP-2 promotes bone development (Maegawa et al., 2007).

#### 4.2.3 Hydrogels with photothermal effects

**FIGURE 7:**
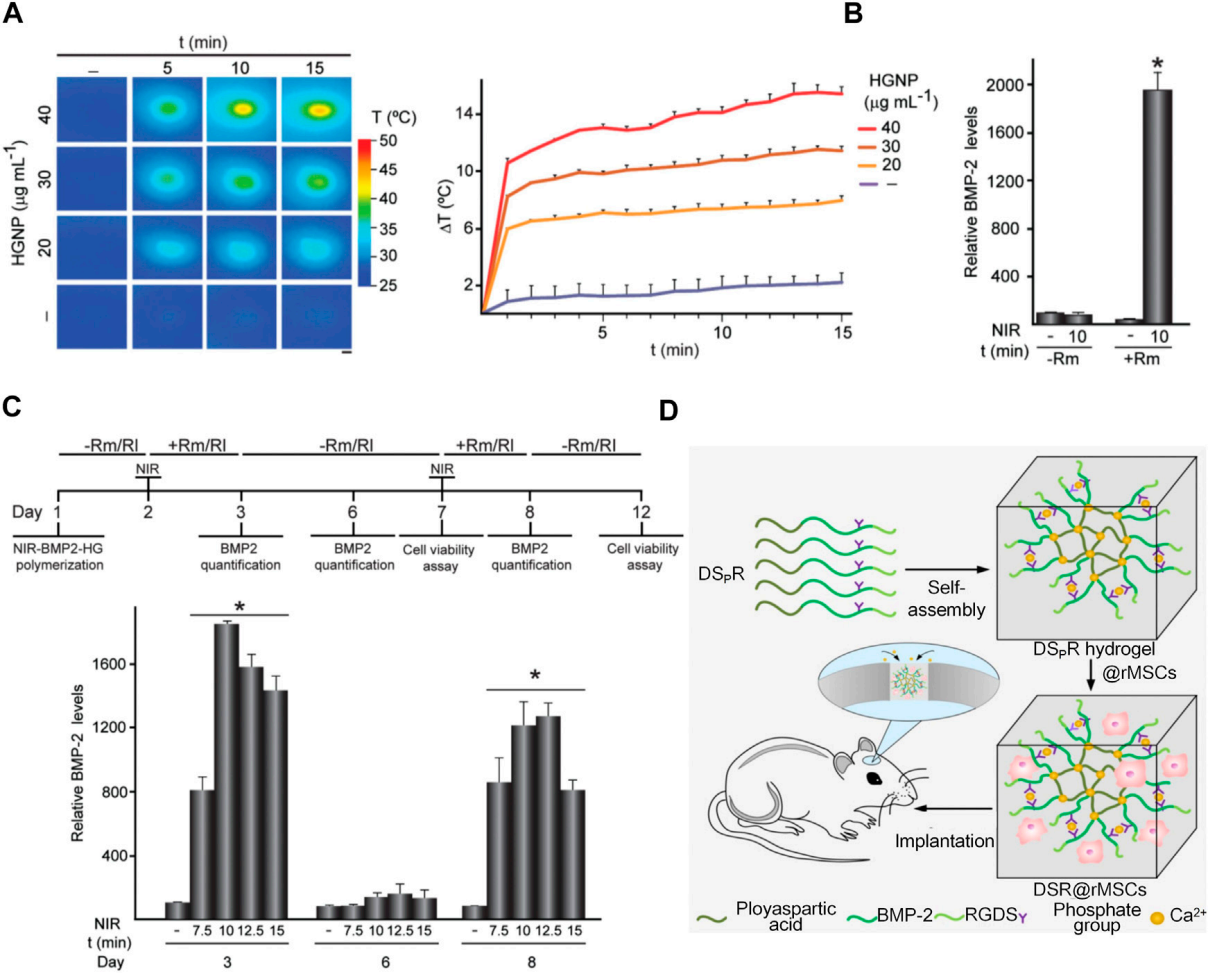
Application of hydrogels with photothermal effects and synthesis of biometic self-assembling peptide hydrogels. **(A)**NIR-BMP-2-HG, polymerized with the indicated concentrations of HGNP, incubated for 1 day, and then irradiated with NIR laser for the indicated time. Infrared thermal image (left). The figure shows the mean +SD value of the maximum temperature rise detected during NIR irradiation (right), *n* = 3. Scale bar = 1 mm. (Pensak et al., 2015; Sanchez-Casanova et al., 2020)(Reprinted with permission) **(B)**BMP-2 concentration in media conditioned with 30 μg ml-1 HGNP-polymerized NIR-BMP-2-HG. (Pensak et al., 2015; Sanchez-Casanova et al., 2020)(Reprinted with permission) **(C)**NIR-BMP-2-HG, aggregated with 30 μg ml-1 HGNP, irradiated by NIR in the presence of 10 nM rapamycin (Rm) or 100 nM rapalog AP21967 (R1). Timeline protocol for NIR-BMP-2-HG preparation, NIR irradiation of hydrogels (NIR), in the absence (-Rm/Rl) or presence (+Rm/Rl) of rapamycin or rapalog and analytically determined cultivated under circumstances. On days 3, 6, and 8, the medium was collected and replaced with fresh medium lacking dimers. Histograms show BMP-2 concentrations on days 3, 6, and 8 in NIR-BMP-2-HG-conditioned media that were NIR-irradiated in the presence of Rm for the indicated times (Pensak et al., 2015; Sanchez-Casanova et al., 2020)(Reprinted with permission). **(D)**Illustration of a 3D bioactive gel scaffold of self-assembled DSpR oligopeptides for repairing rat calvarial defects. (Quan et al., 2019)(Reprinted with permission).

Two main types of hydrogels with photothermal effects, such as inorganic and organic material hybrid hydrogels, are available. Inorganic materials include carbon nanotubes (Deng et al., 2019), gold nanoparticles (Mauck et al., 2006; Matai et al., 2020; Liao et al., 2021), reduced graphene oxide (rGO) (Li et al., 2018; Lima-Sousa et al., 2020), platinum nanoparticles, BP, Au Pds, and TiO2 (15), while organic materials include dopamine (DA) and indocyanine green (ICG). DA has excellent photothermal conversion and adhesion ability, and ICG has excellent photothermal conversion efficiency. While studying the mechanism of osteogenesis, Sanchez’s team fabricated a fibrin/GNPs hydrogel and successfully embedded C3H-BMP-2 high cells into the hydrogel (Figure 7A). Under the simultaneous action of near-infrared irradiation and rapamycin-induced heat treatment, C3H-BMP-2 high cells are stimulated to synthetic BMP-2 for promoting osteogenesis (Figures 7B,C). BMP-2 promotes the differentiation of MSCs into osteoblasts and promotes the role of MSCs in bone defect areas (Pensak et al., 2015; Sanchez-Casanova et al., 2020).

#### 4.2.4Biomimetic self-assembling peptide hydrogels

In the past decade, research has increasingly focused on self-assembling peptide hydrogels used in osteogenesis; Self-assembling peptides of various structures can be fabricated into scaffolds to culture cells and promote bone regeneration. (Sargeant et al., 2012). Quan’s team developed a 3D bioactive scaffold from self-assembling oligopeptides of D9 KIPKASSVPTELSAIS RGDS (DSR) and D9 KIPKASS(p)V PTELSAIS RGDS (DSpR) and included bone morphogenetic protein-2 biomimetic peptide (BMPMP) as a potent osteoinductive cytokine (Figure 7D), polyaspartic acid (D9) as an organic template, calcium chelator, and RGDs as cell adhesion factors that synergistically promote bone regeneration. RGDs and BMPMP promoted the expression of osteogenic genes and accelerated differentiation of MSCs into osteoblasts (Quan et al., 2019).

#### 4.2.5 Conductive hydrogels

Conductive materials in hydrogels have been shown to increase osteoconductivity and mechanical strength. Gold nanoparticles (GNPs) can promote the osteogenic differentiation of MSCs, which are the most valuable substance for bone regeneration. Heo al. used GNPs to synthesize biodegradable hydrogels to promote osteogenesis (Heo et al., 2014). In animal experiments, conductive GNP hydrogels significantly increased osteoblastic activity, proliferation, and bone formation. Conductive fibers were incorporated into hydrogels using graphene nanoparticles and polyaniline to increase their elastic modulus, roughness, and electrical conductivity (Khorshidi and Karkhaneh, 2018). Ezazi designed a bone hydrogel and its main composition are hydroxyapatite, gelatin, and mesoporous silica. Incorporating PPy macromolecules into this hydrogel can provide electrical conductivity; PPy-containing supports exhibit superior mechanical properties than non-conductive supports (Ezazi et al., 2018).

### 4.3 Osteogenic mechanisms associated with drug delivery

Subset 3 covered research on drug delivery in the field of BRMs. From 2002 to 2011, researchers focused on applying three-dimensional scaffolds to drug delivery in bone tissue engineering. After 2012, the research hotspots were mainly the following: 1) Bone tissue engineering and growth factor delivery; 2) Nanobiomaterials used as delivery systems to promote osteogenesis *in vivo*; 3) the potential of *in vivo* exosomal delivery in articular cartilage regeneration, and 4)Autologous platelet-rich fibrin as a drug for bone regeneration.

#### 4.3.1 Application of three-dimensional scaffolds in drug delivery

From 2002 to 2011, widely developed three-dimensional bioactive scaffolds were studied as potential delivery systems for therapeutic drugs that promote bone repair. The main scaffold materials include ceramics, polymers, and composites. As a synthetic bioactive ceramic, CaP (HA, α-tricalcium phosphate (TCP)) has structural and chemical properties similar to the inorganic components of bone and thus has been widely studied as a scaffold material (Karageorgiou and Kaplan, 2005). The rapid development of mesoporous inorganic materials has accelerated the development of composite materials with drug delivery and osteogenic capabilities. Natural polymers, including collagen and polysaccharides (e.g., alginate, hyaluronic acid, and chitosan), have excellent biocompatibility and promote osteogenesis (Mi et al., 2002; Shanmugasundaram et al., 2006; Aoyagi et al., 2007). Composite scaffolds have combined properties of biodegradable polymers and bioactive materials for osteogenesis, which are essentially composite materials with improved mechanical properties. Rezwan K. showed that bioactive inorganic particles such as HA, bioglass, or tricalcium phosphate form strong bonds throughout the growing carbonate HA layer, thereby inducing an efficient interaction between the scaffold and the surrounding bone tissue (Rezwan et al., 2006).

#### 4.3.2 Bone tissue engineering and growth factor delivery

Osteoinductive growth factors such as transforming growth factors (TGF-β) have long been shown to promote bone healing and regulate osteogenesis. Behavioral potentials include recruitment, migration, adhesion, proliferation, and differentiation (Termaat et al., 2005; Bessa et al., 2008; Khojasteh et al., 2013). BMP-2, BMP-6, and BMP-9 can trigger MSC differentiation to stimulate local bone regeneration in osteoblasts (Tong et al., 2019). The delivery of GF relies on polymer scaffolds or composite scaffolds; Linh et al. bound the natural collagen polymers and BMP-2 to the surface of porous HAp scaffolds and found that the composite scaffolds showed higher compressive strength (50.7 MPa) than the HAp scaffolds (45.8 MPa) (Linh et al., 2020). The delivery system produced using this scaffold can effectively induce osteogenic differentiation of adipose-derived stem cells (Walsh et al., 2019).

#### 4.3.3 Nanobiomaterials for bone tissue engineering

Nano-hydroxyapatite (nHA) is widely used as a drug delivery vehicle because of its chemical and structural similarities to bone minerals (Teotia et al., 2017). Several studies have shown that the osteogenic properties of nHA can be improved when combined with other bioactive molecules or drugs. Curtin et al. found that defects implanted with nHA scaffolds containing only BMP-2 or both BMP-2 and bFGF showed higher rates of new bone formation than defects implanted with nHA scaffolds alone (Curtin et al., 2015; Zaffarin, 2021). Raina’s and Teotia’s teams researched the co-delivery of BMP-2 and zoledronic acid by using nano cement whose principal component is nHA; their results revealed that the nanomaterials combined with BMP-2 accelerated osteogenesis (Tran et al., 2014; Teotia et al., 2017).

#### 4.3.4 Potential of *in vivo* exosomal delivery to promote bone regeneration

**FIGURE 8:**
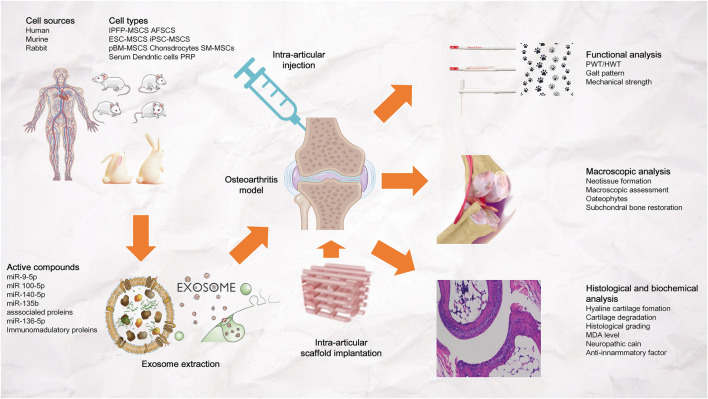
Exosomal bioactive compounds play an important role in cartilage and subchondral bone repair and regeneration. The exosomes were derived from human, murine or rabbit amniotic fluid stem cells (AFSCs), embryonic stem cell-derived mesenchymal stem/stromal cells (ESC-MSCs), induced pluripotent stem cell-derived MSCs (iPSC-MSCs), bone marrow-derived MSCs (BM-MSCs), polydactyly BM-MSCs, synovial membrane-derived MSCs (SM-MSCs), infrapatellar fat pad-derived MSCs (IPFP-MSCs), umbilical cord-derived MSCs (UC-MSCs), chondrocytes, dendritic cells, platelet rich plasma (PRP) and serum. Exosomes were administered to osteoarthritis joints by intra-articular injection or stent implantation.

Exosomes can be derived from various cellsl. However, Most studies choose to extract exosomes from MSCs because MSCs play a significant role in the field of bone tissue repair and osteogenesis (Maas et al., 2017; Cai et al., 2020) (Figure 8). Direct injection is the most widely used delivery method, while the use of scaffolds for exosomal delivery is relatively less studied. Liu et al. (2017b) found that combining exosomes with biomaterials (such as hydrogels) can produce a synergistic effect. Furthermore, they found that compared with *in situ* hydrogel gel implantation and iPSC-MSC-derived exosome injection, iPSC-MSC-derived exosomes implanted into *in situ* hydrogels are more effective in promoting cartilage regeneration (Figure 8).

#### 4.3.5 Autologous platelet-rich Fibrin for osteogenesis

Platelet-rich fibrin (PRF), which can be extracted from a person’s blood, is an easily produced autologous material that promotes wound healing and tissue regeneration. Platelet concentrates also contain growth factors and host immune cells that promote osteogenesis and repair. I-PRF, a recently introduced platelet concentrate, is a liquid injectable PRF that can be added to drugs and drug delivery systems before coagulation (Kour, 2018). A-PRF, on the other hand, is a PRF prepared in the form of a clot; in the A-PRF prepared by Choukroun’s team, more neutrophils are included in the white blood cell count, ensuring their ability to promote the anti-inflammatory state of macrophages, as well as tissue regeneration and angiogenesis (Cabaro et al., 2018; Caruana et al., 2019). PRF can be used as a carrier and can also be combined with other materials to play a role. In one study, freshly pelleted lyophilized platelet-rich fibrin (GL-PRF) was incorporated into polyvinyl alcohol (PVA) hydrogels for sustained release of up to 9 days from GL-PRF/PVA scaffolds. This finding is important because the controlled release of growth factors promotes bone tissue healing and osteogenesis (Xu et al., 2018).

### 4.4 Three-dimensional printing technology

Three-dimensional printing is an essential component of additive manufacturing (AM). Compared with conventional techniques, 3D printing can provide better therapeutic effects and more optimized material properties in clinical applications (Raisian et al., 2017; Simoneti et al., 2022). Several commonly used 3D printing technologies are as listed: 1) Fused deposition modeling (FDM), in which the wire-like hot-melt material is delivered to the hot-melt printing nozzle through a wire feeding mechanism, and the material is heated to a molten state in the nozzle. Under the control of a computer, the nozzle follows the shape contour of the part and the trajectory such that it is deposited in the desired location and then solidifies. (101-102) It can not only improve the biocompatibility and osteoconductivity of scaffolds but can also print personalized scaffolds with different porosities and pore sizes to adapt to the growth and differentiation of stem cells (Gremare et al., 2018; Tian et al., 2020); 2) Stereolithography (SLA), which uses photocurable resins to prepare printed structures. In the SLA equipment, the bottom of the structure is formed by polymerization on the top surface of a moving platform, and the thin layers are aggregated into a two-dimensional pattern drawn by a guided laser beam. Subsequently, the fabrication platform lowers the pattern on top of the upper layer of the polymer to form the desired structure (Su et al., 2021). Studies have shown that the surface modification of SLA implants with Sr nanostructures has a favorable effect on osteoblast function, thereby enhancing osseointegration outcomes (Choi and Park, 2018). 3) PolyJets are similar to FDM and SLA; they work by printing parts one layer at a time using an extruder nose, depositing tiny droplets of a selected photopolymer material on a bed, which is then cured with UV light. Researchers added a polydopamine (PDA)/hydroxyapatite (HA) coating to printed MED610 subjects and found that the PDA/HA coating improved scaffold stiffness, biocompatibility, and osteogenic differentiation potential (Chen et al., 2019; Su et al., 2021).

### 4.5 Citrate for the development of biomimetic composites

In addition to the materials outlined above, citrate is also frequentlyapplied in the development of biomimetic composites for osteogenesis. CBPBHA composites are synthesized based on cross-linked urethane doped polyester (CUPE), polyoctane citrate (POC), and hydroxyapatite (HA). The main synthesis steps are as follows: 1) POC and CUPE premixes were combined to synthesis a homogeneous citrate-based polymer blend (CBPB); 2) various CBPBs were mixed with HA and incubated in Teflon preheated to 50°C to aid solvent evaporation; 3) then, the composites were post-polymerized for 5 days to synthesize cross-linked CBPBHA-X composites (Tran et al., 2014). Richard T et al. implanted CBPBHA-100 and CBPBHA-90 cylindrical composites into the lateral femoral condyle of rabbit knees. Six weeks after implantation, micro-CT images clearly showed the complete fusion of the implant with the surrounding new bone tissue (Tran et al., 2014). Studies have also shown that citrate-based materials can fuel bone regeneration by regulating metabolism to fuel human stem cells. Ma et al. (2018) investigated the expression of the citrate plasma membrane transporter SLC13a5 before and after the osteogenesis of hMSCs. SLC13a5 was found to be the most expressed in undifferentiated and early differentiated hMSCs, and gradually decreased after 4 days of differentiation. Importantly, the addition of the SLC13a5 inhibitor PF06761281 abolished the citrate-induced increase in alkaline phosphatase (ALP) production. Thus, citrate can enhance the bone phenotype through SLC13a5 (Huard et al., 2015).

**FIGURE 9:**
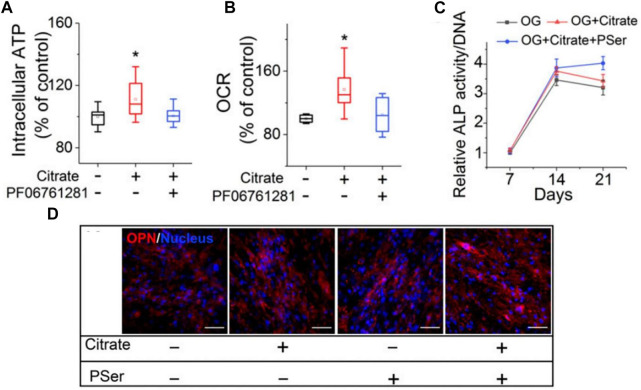
Citrate promotes osteogenesis by regulating metabolism. **(A)**Intracellular ATP assay. **(B)**OCR study. **(C)**ALP production of differentiated hMSCs after 7, 14 and 21 days of differentiation in OG medium supplemented with citrate or citrate and PSer. *n* = 4 biological replicates per group; all data are presented as mean ± SD; *p < 0.05. **(D)**Immunofluorescence staining of OPN (red) with DAPI nuclear counterstain (blue) expression after 21 days of differentiation in OG medium supplemented with citrate, PSer, or both. Plus (+) and minus (−) signs indicate the presence and absence, respectively, of citrate and other specific chemicals in GM/OG medium. (Huard et al., 2015; Ma et al., 2018)(Reprinted with permission).

Citrate can also increase intracellular ATP through metabolic regulation. In a study by Chuying Ma, hMSCs exhibited elevated intracellular ATP levels (Figure 9A) after 24 h of citrate treatment and increased the oxygen consumption rate (OCR) (Figure 9B) (Ma et al., 2018). At the same time, Chuying Ma also found an interesting phenomenon in the experiment: dual treatment with citrate and PSer (a rich functional part of the non-collagen protein (NCP) of the natural bone) had the most obvious bone-promoting effect in the late stage of osteogenesis, and the maintenance is high. Furthermore, in the dual treatment group, the ALP and OPN levels increased until day 21, whereas the ALP levels decreased in the citrate-only treatment and control groups (Park et al., 2011; Ying et al., 2014; Ma et al., 2018) (Figures 9C,D). The research and application of citrate in bone tissue engineering have become a hot topic in recent years. This article focuses only on the tip of the iceberg; the application of such materials in bone tissue engineering is expected to soar in the future.

### 4.6 Limitations

The present study has several limitations. The bibliometric analysis was only based on documents available in WOS. Despite the wide coverage of the WOS database (which is highly similar to that of the Scopus database), we cannot exclude the potential impact on the integrated analysis of the lack of documents from other sources such as the Scopus database. Second, the research topic of bone regenerative biomaterials contains multiple medical disciplines. As only few search terms were used to identify the relevant studies, and only some types of documents were included in the analysis, some potentially valuable documents may have been dismissed.

## Data Availability

The raw data supporting the conclusions of this article will be made available by the authors, without undue reservation.

## Supplementary material

Supplementary Figure.S1 Flowchart for document processing and measurement.
