# Supplementary figures and images for: Bone regeneration materials and their application over 20 years: A bibliometric study and systematic review
Source: Front Bioeng Biotechnol. 2022 Oct 5;10:921092. doi: 10.3389/fbioe.2022.921092 (PMC9581237; doi:10.3389/fbioe.2022.921092)

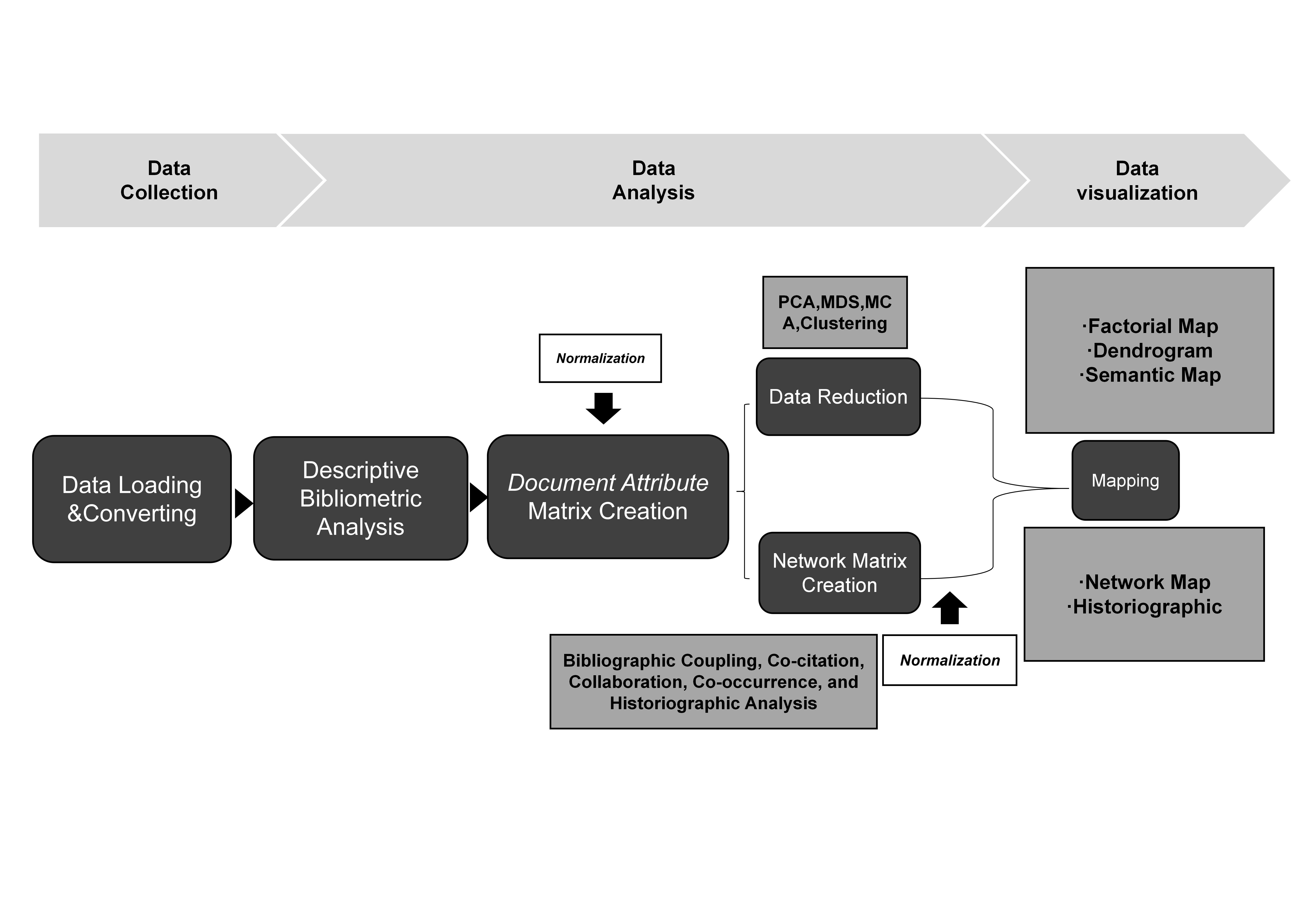

Supplement: Supplementary file 1 [file Image1.JPEG]
